# Control of D-lactic acid content in P(LA-3HB) copolymer in the yeast *Saccharomyces cerevisiae* using a synthetic gene expression system

**DOI:** 10.1016/j.mec.2022.e00199

**Published:** 2022-04-30

**Authors:** Anna Ylinen, Laura Salusjärvi, Mervi Toivari, Merja Penttilä

**Affiliations:** a VTT Technical Research Centre of Finland Ltd, P.O. Box 1000, FI-02044, VTT, Finland; b Department of Bioproducts and Biosystems, School of Chemical Engineering, Aalto University, P.O. Box 11000, FI-00076, AALTO, Finland

**Keywords:** Gene expression, Tet-on, Copolymer, PHA, Doxycycline, Yeast

## Abstract

The fully biobased polyhydroxyalkanoate (PHA) polymers provide interesting alternatives for petrochemical derived plastic materials. The mechanical properties of some PHAs, including the common poly(3-hydroxybutyrate) (PHB), are limited, but tunable by addition of other monomers into the polymer chain. In this study we present a precise synthetic biology method to adjust lactate monomer fraction of a polymer by controlling the monomer formation *in vivo* at gene expression level, independent of cultivation conditions. We used the modified doxycycline-based Tet-On approach to adjust the expression of the stereospecific D-lactate dehydrogenase gene (*ldhA*) from *Leuconostoc mesenteroides* to control D-lactic acid formation in yeast *Saccharomyces cerevisiae*. The synthetic Tet-On transcription factor with a VP16 activation domain was continuously expressed and its binding to a synthetic promoter with eight transcription factor specific binding sites upstream of the *ldhA* gene was controlled with the doxycycline concentration in the media. The increase in doxycycline concentration correlated positively with *ldhA* expression, D-lactic acid production, poly(D-lactic acid) (PDLA) accumulation *in vivo*, and D-lactic acid content in the poly(D-lactate-*co*-3-hydroxybutyrate) P(LA-3HB) copolymer. We demonstrated that the D-lactic acid content of the P(LA-3HB) copolymer can be adjusted linearly from 6 mol% to 93 mol% *in vivo* in *S. cerevisiae*. These results highlight the power of controlling gene expression and monomer formation in the tuning of the polymer composition. In addition, we obtained 5.6% PDLA and 19% P(LA-3HB) of the cell dry weight (CDW), which are over two- and five-fold higher accumulation levels, respectively, than reported in the previous studies with yeast. We also compared two engineered PHA synthases and discovered that in *S. cerevisiae* the PHA synthase PhaC1437_Ps6-19_ produced P(LA-3HB) copolymers with lower D-lactic acid content, but with higher molecular weight, in comparison to the PHA synthase PhaC1Pre.

## Highlights

• P(LA-3HB) monomer structure was adjusted with controlled gene expression.
• Expression of D-lactate dehydrogenase (*ldhA*) was controlled with Tet-On approach.
• Lactic acid content in copolymer P(LA-3HB) was adjusted from 6 mol% up to 93 mol%.
• 5.6% PDLA and 19% P(LA-3HB) of cell dry weight (CDW) were obtained in *S. cerevisiae*.
• *PhaC1437*_*Ps6-19*_ P(LA-3HB) had lower D-lactic acid % than *PhaC1Pre* P(LA-3HB).

## Abbreviations

3HB: 3-hydroxybutyrate
CoA: Coenzyme A
*DLD1*: D-lactate dehydrogenase gene
ENO1cp: ENO1 core promoter
LA: Lactic acid
*ldhA*: Stereospecific D-lactate dehydrogenase gene from *Leuconostoc mesenteroides*
mcl: Medium chain length
Mw: Weight average molecular weight
Mn: Number average molecular weight
Đ: Dispersity
PDLA: Poly(D-lactic acid)
PHA: Polyhydroxyalkanoate
PHB: Poly(3-hydroxybutyrate)
P(LA-3HB): Poly(D-lactate-*co*-3-hydroxybutyrate)
*pTDH3*: *TDH3* gene promoter
scl: Short chain length
SES: Synthetic expression system
sTF: Synthetic transcription factor
Tg: Glass transition temperature
*UBC6*: Ubiquitin-protein ligase gene
VP16: Transcription activation domain from a herpes simplex virus

## 1 Introduction

The global awareness of plastic waste management issues has increased the demand for novel environmentally friendly materials. Native and engineered microorganisms are able to produce biobased, biodegradable, and thermoformable poly(hydroxyalkanote) (PHA) polymers, including poly(3-hydroxybutyrate) (PHB) (Peoples and Sinskey, 1989a; 1989b) and many different short- and medium chain length PHAs (Choi et al., 2020). In addition, protein engineering of few PHA synthases (Jung et al., 2010; Taguchi et al., 2008; Yang et al., 2010) and propionyl-CoA transferases (Prabhu et al., 2012; Taguchi et al., 2008; Yang et al., 2010) has enabled production of poly(D-lactic acid) (PDLA) *in vivo*.

The mechanical properties of some homopolymeric PHAs are rather limited, but tunable by incorporation of other monomers into the polymer chain. For example, the presence of only 15 mol% of D-lactic acid in the poly(D-lactate-*co*-3-hydroxybutyrate) copolymer (P[LA-3HB]) increases the polymer flexibility over 8-fold and decreases the melting temperature by 10 °C (Yamada et al., 2011), in comparison to PHB. The lower melting temperature facilitates material processing below the thermal decomposition temperature. The increased flexibility and lower melting temperatures are not limited to P(LA-3HB) copolymer, but similar phenomena are observed also with PHA copolymers containg 3-hydroxypropionate and 4-hydroxybutyrate monomers (Doi et al., 1990; Li et al., 2010; Meng et al., 2012). In fact, a recent PHA modelling study, focusing on predicting the glass transition temperature (Tg) of different PHA copolymers, reports that the relative amount of two different monomers is the second most important parameter defining the Tg, after the choice of the monomer (Jiang et al., 2020). These findings emphasise the importance of controlling the monomer ratios in development of the new PHA copolymers.

Production of the P(LA-3HB) copolymer has been studied earlier *in vitro* and *in vivo* in two bacterial strains, *Escherichia coli* and *Corynebacterium glutamicum,* as reviewed in (Choi et al., 2020), and most recently *in vivo* by us in the yeast *Saccharomyces cerevisiae* (Ylinen et al., 2021)*.* In previous studies polymers with D-lactic acid contents from 3 to 30 mol% (Kadoya et al., 2015), 20–50 mol% (Yang et al., 2010), 8–73 mol% (Nduko et al., 2014), and from 55 to 86 mol% (Jung et al., 2010), were obtained by controlling physiological parameters such as aeration and monomer feeding, and by expressing different PHA synthases. In the yeasts *S. cerevisiae* and *Yarrowia lipolytica* physiological parameters, substrate feeding, and changes in β-oxidation were used for controlling monomer content in medium chain length (mcl) and short chain length (scl) PHA copolymers (De Oliveira et al., 2004; Gao et al., 2015; Haddouche et al., 2010, 2011; Poirier et al., 2001; Rigouin et al., 2019; Zhang et al., 2006). However, none of the studied methods is readily applicable for ubiquitous control of production of other monomers *in vivo.* This would be highly important when novel monomers are too expensive, toxic, or unavailable for feeding in sufficient concentrations. We wanted to study tuning of monomer ratios at the level of gene expression to control polymer properties independent of cultivation conditions. So far, this has been studied relatively little and only in bacterial species. Only one study in *E. coli* exploits the possibility to induce gene expression of 3-hydroxybutyrate-CoA (3HB-CoA) related genes with different arabinose concentrations (Wang et al., 2013). Results from that study are however encouraging with a wide range (10–95 mol%) of 3-hydroxypropionate in PHB backbone. The remaining few bacterial studies rely on building of large strain libraries with different promoters or ribosome binding sites, or on CRISPRi based repression of gene expression, and report only smaller monomer ratios of 0–13 mol% or 0–45 mol% (3-hydroxyvalerate, 4-hydroxybutyrate, or 3-hydroxyhexanoate) in PHB backbone (Arikawa et al., 2016; Lv et al., 2015; Tao et al., 2017; Yu et al., 2020).

The PHA production in yeasts has not been studied as widely as in bacterial strains. However, different yeast species offer interesting options for the PHA production being capable of growing on different inexpensive substrates and tolerating acidic conditions (Rigouin et al., 2019; Sandström et al., 2015). In addition, yeasts lack endotoxins and PHA depolymerases present in many bacterial strains. Yeast cellular compartments allow also separation of different precursors to distinct locations, increasing thus possibilities to modify the PHA polymerization process and final monomer compositions. PHA production was first shown in yeasts by introducing a PHA synthase gene *phaC1* from native bacterial PHB producer *Cupriavidus necator* into *S. cerevisiae* (Leaf et al., 1996). Resulting strain accumulated 0.5% PHB of cell dry weight (CDW). PHB accumulation was later increased in *S. cerevisiae* to approximately 9% of CDW by introduction of entire PHB pathway from *C. necator,* including genes for acetyl-CoA acetyltransferase (*phaA*) and acetoacetyl-CoA reductase (*phaB1*) (Carlson and Srienc, 2006), and up to 14–16.4% of CDW when *phaB1* was replaced with gene encoding NADH dependent acetoacetyl-CoA reductase variant from *Allochromatium vinosum* and strains were grown on xylose in anerobic conditions (de Las Heras et al., 2016; Portugal-Nunes et al., 2017). Engineering of PHB and mcl-PHA production in other yeasts has resulted in accumulation of up to 25%, 30%, and 52% PHAs of CDW in yeasts *Y. lipolytica, Pichia Pastoris*, and *Arxula adeninivorans,* respectively (Biernacki et al., 2017; Rigouin et al., 2019; Vijayasankaran et al., 2005). In our previous study we engineered the yeast *S. cerevisiae* for the production of PDLA, PHB, and their copolymer P(LA-3HB) (Ylinen et al., 2021). We obtained D-lactic acid contents of 46–65% in the copolymer P(LA-3HB) by expressing a stereospecific D-lactate dehydrogenase gene (*ldhA*) from *Leuconostoc mesenteroides* from a constitutive *pTDH3* promoter.

Here we studied the possibility to adjust the P(LA-3HB) monomer composition at the gene expression level by regulating the D-lactic acid production *in vivo*. The expression of the *ldhA* gene was adjusted with a doxycycline controlled Tet-On expression system (Kakko et al. in preparation; (Bellí et al., 1998; Gossen et al., 1995; T. Das et al., 2016), which was modified based on our previously constructed synthetic expression system (SES), where the synthetic transcription factor controls expression of a synthetic promoter consisting of a varying number of binding sites followed by a short core promoter (Rantasalo et al., 2018a). The controlled D-lactic acid production inspired us also to study how the D-lactic acid availability affects the PDLA accumulation in the cells. In addition, two engineered PHA synthases and their different copy numbers were compared for their efficiencies in the D-lactyl-CoA polymerization in *S. cerevisiae*.

## 2 Materials and methods

### 2.1 Strains and plasmids

The studied genes, plasmids, and oligos are listed in Table 1 and the yeast strains in Table 2. The parent strain, haploid *S. cerevisiae* strain CEN.PK111-9A (H3892), with *LEU2* and *HIS3* auxotrophies, was kindly provided by Dr. P. Kötter from Institut für Mikrobiologie (J.W. Goethe Universität Frankfurt, Germany). Plasmids were cloned using Gibson Assembly (E2611S, New England BioLabs) and *E. coli* TOP10 cells. The lithium acetate method (Gietz and Schiestl, 2007) was used for all yeast transformations.

**Table 1:** The studied genes, plasmids, and oligos.

Genes		
**Name**	**Description**	**Reference**
*phaA*	Acetyl-CoA acetyltransferase from *Cupriavidus necator*, GenBank KP681582	Sandström et al. (2015)
*phaB1*	Acetoacetyl-CoA reductase from *C. necator,* GenBank KP681583	Sandström et al. (2015)
*ldhA*	Stereospecific D-lactate dehydrogenase (*ldhA*) from *Leuconostoc mesenteroides*	Baek et al. (2016)
*pctMe*	Propionyl-CoA transferase from *Megasphaera elsdenii*, European Nucleotide Archive ERZ1065933	Prabhu et al. (2012)
*phaC1437*_*Ps6-19*_	PHA synthase from *Pseudomonas* sp. MBEL 6–19, with amino acid substitutions E130D, S325T, S477G, and Q481K	Yang et al. (2010)
*phaC1Pre*	PHA synthase from *Pseudomonas resinovorans*, with amino acid substitutions E130D, S325T, S477G, and Q481K	Yang et al. (2011)

**Plasmids**			
**Name**	**Description**	**EasyClone integration locus**	**Reference**
B11841	*pTEF1-pctMe-tCYC*	X-3	This article
B11843	*pTDH3-phaC1Pre-tCYC*	X-4	This article
B11847	*pTDH3-phaC1Pre-tCYC*	XII-5	This article
B11848	*pTDH3-phaC1Pre-tCYC*	XI-3	This article
B11844	*pTDH3- phaC1437*_*Ps6-19*_*-tCYC*	X-4	This article
B11845	*pTDH3- phaC1437*_*Ps6-19*_*-tCYC*	XII-5	This article
B11846	*pTDH3- phaC1437*_*Ps6-19*_*-tCYC*	XI-3	This article
B11849	*pTDH3-phaC1Pre-tCYC*	X-2	This article
B11850	*pTDH3-phaC1Pre-tCYC*	XII-4	This article
B11851	*pTDH3-phaC1Pre-tCYC*	XI-5	This article
B11852	*pTDH3 phaC1437*_*Ps6-19*_*-tCYC*	X-2	This article
B11853	*pTDH3- phaC1437*_*Ps6-19*_*-tCYC*	XII-4	This article
B11854	*pTDH3- phaC1437*_*Ps6-19*_*-tCYC*	XI-5	This article
B11855	*pTEF1-phaA-tENO1-pTDH3-phaB1-tCYC*	XI-I	This article
B9091	*pTDH3-TetR-VP16*, Kl*LEU2* marker from *Kluyveromyces lactis*		(Kakko et al. in preparation)
B11839	gRNA1 (*DLD1*), gRNA sequence CACAGCCAAACATCAAACCG		This article
B11840	gRNA2 (*DLD1*), gRNA sequence ATAGTCTGGTGAGTCCAATG		This article
B11842	60 bp flank to *DLD1*-8BS + *ENOcp-ldhA-tENO2*-60bp flank to *DLD1*		This article

**Oligos**		
**Name**	**Sequence**	**Reference**
UBC6_qPCR_F (1189)	ACTTTCCCGTCTGATTATCCA	Rantasalo et al. (2018b)
UBC6_qPCR_R (1190)	TAATTGATCCTGTCGTGGCT	Rantasalo et al. (2018b)
ldhA_1 qPCR_F	ATGCCATAAGTCTGTACGTCC	This article
ldhA_1 qPCR_R	AAATTCCTTTCCACTCCAGTC	This article
ldhA_2 qPCR_F	AGAGTGGCGATTAACATCCT	This article
ldhA_2 qPCR_R	GGCGTCTATATCCATCAGATTACC	This article

**Table 2:** Yeast strains used in this study.

Strains				
Name	Number	Description	Integrated plasmids	Reference
CEN.PK111-9A	H3892	*S. cerevisiae*, CEN.PK111-9A (*MATa his3-Δ1 URA3 leu2-3,112 TRP1 MAL2-8c SUC2*)		
pTDH3-ldhA	H5513	*S. cerevisiae, CEN.PK102–5B (MATa his3-Δ1 ura3-52 leu2-3,112 TRP1 MAL2-8c SUC2, DLD1::pTDH3-ldhA*)		Ylinen et al. (2021)
pTDH3-ldhA-2u_pTDH3_ phaC1Pre	H5520	H5513 with 2μ plasmid B9664 (*pTEF1-pctMe-tENO1-pTDH3-phaC1Pre-tSSA1-LEU2*)		Ylinen et al. (2021)
pTEF1-pctMe	H5720	H3892 with *pTEF1-pctMe*	B11841	This article
pTDH3-tetR-VP16	H5721	H3892 with LEU2, *pTDH3-tetR-VP16*	B9091	This article
pTEF1-pctMe-TetOn-ldhA	H5723	H3892 with *LEU2, pTDH3-tetR-VP16, pTEF1-pctMe*, *DLD1::TetOn-ldhA*	B9091, B11841, B11842	This article
phaC1Pre_1x	H5724	H5723 with *pTDH3-phaC1Pre*	B11843	This article
phaC1437_1x	H5725	H5723 with *pTDH3- phaC1437*_*Ps6-19*_	B11844	This article
phaC1Pre_3x	H5726	H5723 with 3x *pTDH3-phaC1Pre*	B11843, B11847, B11848	This article
phaC1437_3x	H5728	H5723 with 3x *pTDH3- phaC1437*_*Ps6-19*_	B11844, B11845, B11846	This article
phaC1Pre_6x	H5727	H5723 with 6x *pTDH3-phaC1Pre*	B11849, B11850, B11851	This article
phaC1437_6x	H5729	H5723 with 6x *pTDH3- phaC1437*_*Ps6-19*_	B11852, B11853, B11854	This article
phaC1Pre_3HB	H5730	H5726 with *pTEF1-phaA, pTDH3-phaB1*	B11855	This article
phaC1437_3HB	H5731	H5728 with *pTEF1-phaA, pTDH3-phaB1*	B11855	This article

**Fig. 1:**
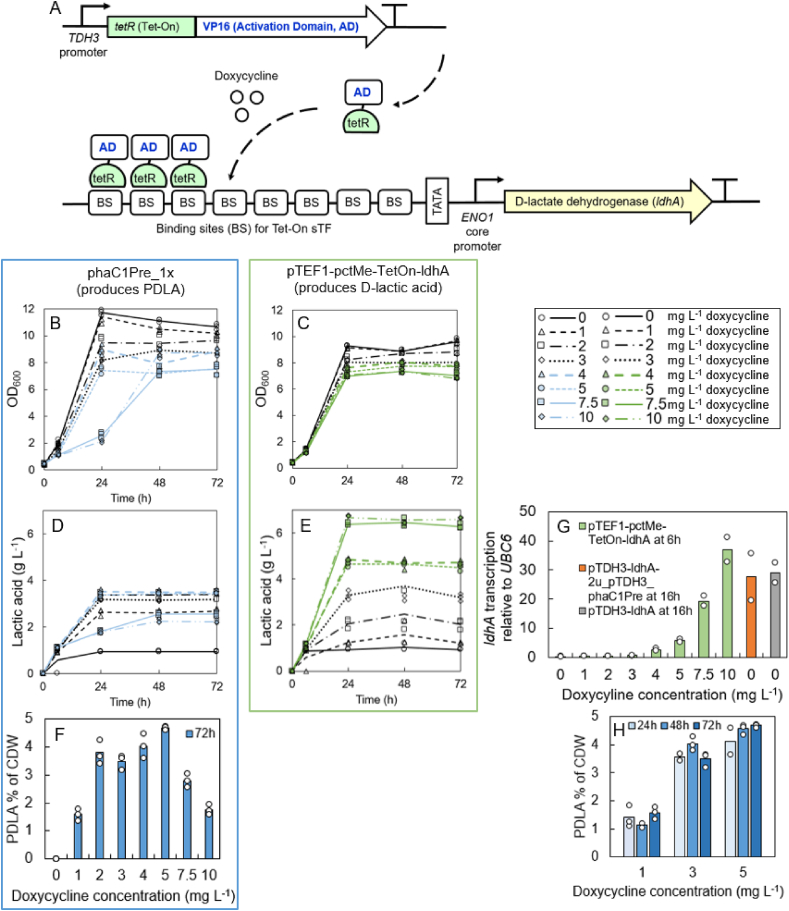
A: Schematic presentation of the Tet-On method used in this study to control expression of the D-lactate dehydrogenase gene (*ldhA)* from *L. mesenteroides.* Increase in doxycycline concentration results in higher binding of the synthetic transcription factor and enhanced *ldhA* expression. B–H: The results of the cultivation of the PDLA strain phaC1Pre_1x and its control strain pTEF1-pctMe-TetOn-ldhA for 72 h with 20 g L^−1^ glucose and with doxycycline concentrations of 0, 1, 2, 3, 4, 5, 7.5, and 10 mg L^−1^. B, C: Growth as OD_600_, D, E: D-lactic acid production (g L^−1^), F, H: PDLA accumulation as % of CDW (strain PhaC1Pre_1x). The values represent averages of three biological replicates. Individual data points are presented with triangles, squares, diamonds, or circles. G: The *ldhA* transcription relative to expression of the ubiquitin-protein ligase gene (*UBC6*) in the strain pTEF1-pctMe-TetOn-ldhA at 6 h, and the control strains pTDH3-ldhA-2u-pTDH3_phaC1Pre and pTDH3-ldhA at 16h. The circles represent average values of the three biological replicates obtained with two oligo pairs (Table 1).

The endogenous D-lactate dehydrogenase gene (*DLD1*) of the strain CEN.PK111-9A was deleted with simultaneous integration of codon optimized, stereospecific D-lactate dehydrogenase from *L. mesenteroides* (*ldhA*) (Baek et al., 2016; Ylinen et al., 2021). Deletion was carried with CRISPR/Cas9 method using two gRNA plasmids B11839 and B11840 presented in Table 1. The *ldhA* gene was expressed either under a constitutive *pTDH3* promoter or a doxycycline controllable Tet-On system (Fig. 1) (Bellí et al., 1998; Gossen et al., 1995; T. Das et al., 2016) containing Tet-On synthetic transcription factor (sTF) described by Kakko et al. (in preparation), eight bindig sites for the sTFs (Rantasalo et al., 2018a), and an *ENO1* core promoter (*ENO1cp*) (Rantasalo et al., 2018a). The *ENO1cp* is a minimal sequence required for initiation of the transcription and it contains binding sites for common transcription factors, which do not adjust the strenght of the transcription, but form a complex with the DNA polymerase. The Tet-On synthetic transcription factor (sTF) construct from plasmid B9091 (Kakko et al., in preparation) was integrated into the *LEU2* locus of *S. cerevisiae* CEN.PK111-9A, resulting in the strain pTDH3-TetR-VP16*.* This strain was used as parent strain for constructing control strain pTEF1-pctMe-TetOn-ldhA and polymer strains listed in Table 2 (H5724–H5731). Propionyl-CoA transferase gene *pctMe* from *Megasphaera elsdenii,* acetyl-CoA acetyltransferase gene *phaA* and acetoacetyl-CoA reductase gene *phaB1* from *C. necator*, and PHA synthase genes *phaC1437*_*Ps6-19*_ and *phaC1Pre* (Table 1) were cloned into EasyClone integration vectors (Jessop-Fabre et al., 2016), linearized with NotI restriction enzyme (FD0596, Thermo Scientific), and integrated to the yeast strains using CRISPR/Cas9 technology and EasyClone gRNA vectors (Jessop-Fabre et al., 2016) for the selection.

### 2.2 Culture conditions and analytical methods

*E. coli* was grown in Luria–Bertani medium containing either ampicillin (100 μg/ml) or kanamycin (50 μg/ml). The yeast strains were grown in 50 ml of synthetic complete media supplemented with 20 g L^−1^ glucose and 0–10 mg L^−1^ doxycycline in three replicates for 24–72 h at 30 °C with 220 rpm shaking. The cell growth, pH, and metabolite production were analyzed daily as described previously (Ylinen et al., 2021). The cell growth was followed as optical density (OD_600_), or as CDW. When necessary, the OD_600_ values for the strain phaC1Pre_1x were converted to CDW with a linear correlation function (OD_600_ = 3.9822* CDW+ 0.9895, R^2^ 0.98) derived from another experiment where the same strain was grown in similar conditions (Supplemental Fig. S1).

#### 2.2.1 Cell growth study in bioscreen C equipment

The cell growth with doxycycline concentrations of 0, 1, 5, and 10 mg L^−1^ was followed every 10 min in Bioscreen C equipment in multiwell plates at 30 °C using 200 μl total volume of the media and a starting OD_600_ of approximately 0.10–0.11. The starting OD_600_ was measured with VitroSpec 2100 Pro equipment (Amersham Biosciences), but later the growth was followed with the Bioscreen spectorofotometer directly from the multiwell plates. The samples were analyzed in three technical and biological replicates. The outer wells were filled with water to decrease the media evaporation from the sample wells.

#### 2.2.2 Polymer extraction and analysis

The polymers were analyzed as described previously (Ylinen et al., 2021). The cells were washed with distilled water three times and lyophilized over night. The quantity and the composition of the accumulated polymers were analyzed directly from the lyophilized cells with gas chromatography–mass spectrometry (GC-MS). The polymers were extracted from the lyophilized cells by boiling the cells in 95 °C chloroform for 3 h followed by filtration through 0.45 μm PTFE filters and two non-solvent precipitation washing steps. The phospholipids were removed with methanol and the neutral lipids with diethyl ether. The molecular weights of the extracted polymers were analyzed with a chloroform based size exclusion chromatography (SEC).

#### 2.2.3 Transcriptional analysis

The transcription analysis was carried as described previously (Rantasalo et al., 2018a). Two oligo pairs (Table 1) were used for detecting 233 and 258 bp PCR products of the gene *ldhA*. The ubiquitin-protein ligase gene *UBC6*, with high transcriptional stability (Teste et al., 2009), was used as a reference gene for normalization of the expression levels.

## 3 Results

### 3.1 Regulation of *ldhA* expression and its effect on D-lactic acid production and PDLA accumulation

To control the D-lactic acid production in *S. cerevisiae*, the expression of stereospecific D-lactate dehydrogenase gene (*ldhA)* from *L. mesenteroides* (Baek et al., 2016) was adjusted with doxycycline controlled Tet-On method (Fig. 1) (Bellí et al., 1998; Gossen et al., 1995; T. Das et al., 2016). The system contained constitutively expressed sTF TetR-VP16 (Kakko et al. in preparation) and eight binding sites for TetR-VP16 upstream of the core promoter derived from the *ENO1* promoter (*ENO1cp)* (Rantasalo et al., 2018a)*,* and the *ldhA* gene. Eight binding sites were chosen based on previous studies (Bellí et al., 1998; Rantasalo et al., 2016) to enable wide range of different expression levels from low to high in the tunable Tet-On expression system. The binding of TetR-VP16 to the binding sites was controlled with the concentration of the doxycycline in the media. The endogenous D-lactate dehydrogenase gene (*DLD1*) was deleted to prevent the oxidation of produced D-lactic acid and thus to increase the D-lactic availability for polymerization. The strain was further engineered by integration of propionyl-CoA transferase gene *pctMe* from *M. elsdenii* with constitutive *pTEF1* promoter (strain pTEF1-pctMe-TetOn-ldhA) and by integration of an engineered PHA synthase gene *phaC1Pre* from *Pseudomonas resinovorans* carrying four amino acid substitutions, E130D, S325T, S477G, and Q481K, with constitutive *pTDH3* promoter (strain phaC1Pre_1x). The PDLA and P(LA-3HB) pathways are presented in Supplemental Fig. S2. The strains expressing only *tetR-VP16*, *pctMe*, or *ldhA* (Table 2) were used as controls. The constructed strains were first grown with different doxycycline concentrations in the Bioscreen C instrument in multiwell plates for 15 h. The expression of *pctMe* or *tetR-VP16* under constitutive promoters did not affect cell growth (Supplemental Table S1, Supplemental Fig. S3). The highest doxycycline level of 10 mg L^−1^ had only very minor effect on the specific growth rate and the final OD_600_ of the control strains *pTEF1*-*pctMe* or the parent strain CEN.PK111-9A. The expression of the *ldhA* gene from the *TDH3* promoter or from the Tet-On construct with 10 mg L^−1^ doxycycline decreased the growth rate by approximately 50% in comparison to the parent strain CEN.PK111-9A.

**Fig. 2:**
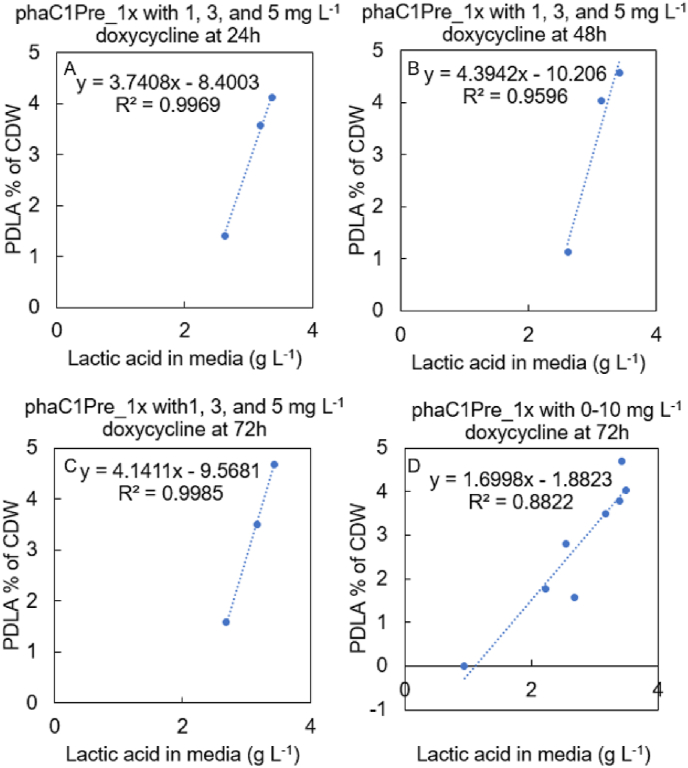
The correlation of the D-lactic acid concentration produced to the media (g L^−1^) to the PDLA accumulation % of CDW in the strain phaC1Pre_1x, in which the *ldhA* expression was controlled with 1, 3, and 5 mg L^−1^ doxycycline (A–C) or with 0, 1, 2, 3, 4, 5, 7.5, and 10 mg L^−1^ doxycycline (D). The samples were analyzed at 24 h (A), 48 h (B), and at 72 h (C, D).

To measure the D-lactic acid and the PDLA production at the different expression levels of the *ldhA*, the PDLA strain (strain phaC1Pre_1x) and the corresponding control strain without PHA synthase (strain pTEF1-pctMe-TetOn-ldhA*)* were grown with 20 g L^−1^ glucose and doxycycline concentrations of 0, 1, 2, 3, 4, 5, 7.5, and 10 mg L^−1^ in 50 ml volume. The results demonstrated that it was possible to reduce the extracellular D-lactic acid production from 6.5 to 1 g L^−1^ by decreasing the doxycycline concentration from 7.5 to 0 mg L^−1^ (Fig. 1E, Supplemental Fig. S5). The *ldhA* transcription was downregulated accordingly when doxycycline was decreased (Fig. 1G). The accumulation of intracellular PDLA correlated linearly with the production of extracellular D-lactic acid (Fig. 2). The time profile of intracellular PDLA accumulation was followed with strain phaC1Pre_1x grown with 1, 3, and 5 mg L^−1^ doxycycline. The majority of the PDLA polymer accumulated already within the first 24 h (Fig. 1H). Only small 13% increase in PDLA was observed from 24 h to 72 h with 1 and 5 mg L^−1^ doxycycline concentrations. The maximum 4.7% PDLA percentage of CDW was obtained at 72 h with 5 mg L^−1^ doxycycline (Fig. 1F). The PDLA production was also calculated as mg L^−1^. Result showed that from 24 h to 72 h the PDLA titer increased only 5% and 15% with 3 mg L^−1^ and 5 mg L^−1^ doxycycline, respectively (Supplemental Fig. S6).

The mRNA levels between the constitutive *pTDH3* promoter and doxycycline-controlled Tet-On system were also compared. The *ldhA* expression from the constitutive *pTDH3* promoter was measured from the control strain pTDH3-ldhA and from the strain used in our previous study pTDH3-ldhA-2u-pTDH3_phaC1Pre (H5520) (Ylinen et al., 2021). Their expression was on average 24% lower than expression from Tet-On promoter induced with 10 mg L^−1^ doxycycline, but 44% higher compared to the Tet-On promoter with 7.5 mg L^−1^ doxycycline.

### 3.2 Comparison of the two PHA synthases and their expression levels for PDLA production

**Fig. 3:**
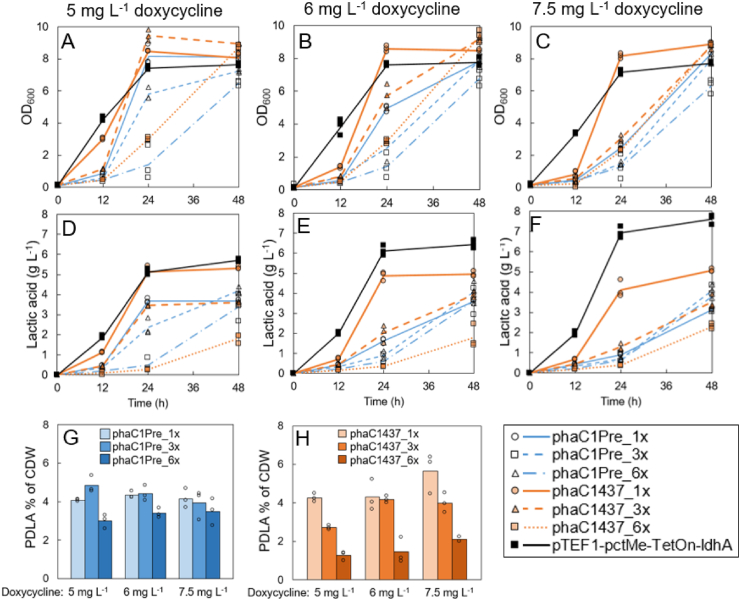
Comparison of the strains with the engineered PHA synthases, phaC1Pre and phaC1437_Ps6-19_, and their expression from one, three, or six copies the corresponding genes. The *ldhA* expression was controlled with 5, 6, and 7.5 mg L^−1^ doxycycline. A–C: Growth as OD_600_, D–F: D-lactic acid production (g L^−1^), G–H: PDLA accumulation as % of CDW. The values represent averages of three biological replicates. The individual data points are presented with circles, squares, and triangles.

The PHA synthases phaC1Pre from *P. resinovorans* (Yang et al., 2011) and phaC1437_Ps6-19_ from *Pseudomonas* sp. MBEL 6–19 (Yang et al., 2010) were compared for their D-lactic acid polymerization efficiencies in *S. cerevisiae*. While both synthases carry the same amino acid substitutions E130D, S325T, S477G, and Q481K, their amino acid identity is only approximately 80% (Yang et al., 2011). One, three, or six copies of each PHA synthase gene under the *pTDH3* promoter were integrated into the control strain pTEF1-pctMe-TetOn-ldhA (Table 2). In cultivations of the resulting strains, the *ldhA* expression was adjusted with doxycycline concentration of 5 mg L^−1^ that resulted in the highest PDLA production in the previous experiment, and in addition with two higher concentrations of 6.0 and 7.5 mg L^−1^. Cells were grown with 20 g L^−1^ glucose for 48 h. When the PHA synthase copy number increased from one to three or six copies, most of the strains showed delay in growth, glucose consumption, production of acetate and D-lactic acid, and PDLA accumulation (Fig. 3, Supplemental Fig. S7). Only the strain with three copies of the PHA synthase gene *phaC1Pre* (strain phaC1Pre_3x) (Fig. 3G and H) accumulated more PDLA than strain with one copy of the corresponding gene (phaC1Pre_1x), but this improvement can be explained by the higher D-lactic acid production of the strain phaC1Pre_3x (Supplemental Fig. S8D). The strain with one copy of PHA synthase gene *phaC1437*_*Ps6-19*_ (strain phaC1437_1x) accumulated the most PDLA of all strains studied, 5.6% of CDW, when *ldhA* expression was controlled with the highest doxycycline concentration, 7.5 mg L^−1^. This strain produced also the most extracellular D-lactic acid, 5.1 g L^−1^, and grew to the highest OD_600_ in 24 h, when compared to other strains grown with 7.5 mg L^−1^ doxycycline. Cultivation of this strain resulted to the lowest medium pH and highest ethanol and acetate production at 24 h (Supplemental Fig. S7). When the strain phaC1437_1x was controlled with lower doxycycline concentrations of 5.0 and 6.0 mg L^−1^, less PDLA accumulated, only 4.3% of CDW. The extracellular D-lactic acid formation and intracellular PDLA accumulation showed clear positive correlation also in this experiment with the strain expressing the PHA synthase *phaC1437*_*Ps6-19*_ (Supplemental Fig. S8).

### 3.3 Effect of the regulation of *ldhA* expression on the D-lactic acid content of the P(LA-3HB) copolymer

**Fig. 4:**
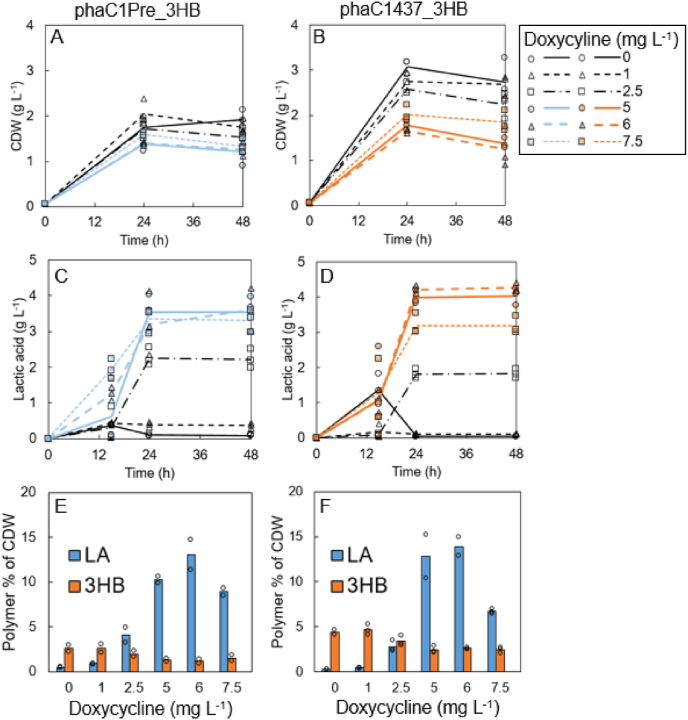
Comparison of the copolymer strains phaC1Pre_3HB and phaC1437_3HB. The expression of the *ldhA* was controlled with the Tet-On system using either 0, 1, 2.5, 5, 6, or 7.5 mg L^−1^ doxycycline. A–B: The cell growth as cell dry weight; C–D: Production of D-lactic acid (g L^−1^). E–F: Accumulation % of the P(LA-3HB) of CDW at 48 h, bars represent fraction of each monomer of CDW (g g^−1^). The values represent averages of two or three biological replicates. The individual data points are presented with circles, triangles, or squares.

The Tet-On enabled regulation of the *ldhA* expression was exploited for controlling the D-lactic acid content in the copolymer P(LA-3HB). The 3-hydroxybutyryl-CoA pathway genes *phaA* and *phaB1* from *C. necator* were integrated with the *pTEF1* and *pTDH3* promoters, respectively, into the PDLA strains phaC1Pre_3x and phaC1437_3x. These parental strains with three copies of the PHA synthase genes were chosen because of their high PDLA accumulation levels with doxycycline concentration of 6 mg L^−1^ in the previous experiment (Fig. 3). The resulting strains phaC1Pre_3HB and phaC1437_3HB were grown for 48 h with the doxycycline range from 0 to 7.5 mg L^−1^. The results demonstrated that by controlling the *ldhA* gene expression with Tet-On system it is possible to adjust the D-lactic acid fraction in the copolymer P(LA-3HB). Linear range of D-lactic acid proportions from 6 to 93 mol% of the P(LA-3HB) copolymer was obtained (Fig. 4E and F, Table 3). Increase of the D-lactic acid production in the range of 1–6 mg L^−1^ doxycycline resulted in decrease in ethanol, acetate, and biomass formation in the strains (Fig. 4, Supplemental Fig. S9). The expression of PHA synthase phaC1Pre resulted in higher D-lactic acid content in the polymer with all the used doxycycline concentrations in comparison to the expression of the phaC1437_Ps6-19_ enzyme. However, the P(LA-3HB) yield as mg g^−1^ glucose utilised was 30–140% higher with strain phaC1437_3HB in comparison to the strain phaC1Pre_3HB (Table 3). The highest titer of 232 mg L^−1^ and the highest total copolymer accumulation of 19.0% of CDW with the D-lactic acid proportion of 88 mol% were obtained with the strain phaC1437_3HB using 6 mg L^−1^ doxycycline. The slightly higher D-lactic acid proportion of 93 mol%, but lower polymer accumulation level of 14.2% of CDW was obtained with the strain phaC1Pre_3HB controlled with the same 6 mg L^−1^ doxycycline concentration. There was a clear positive correlation between the produced extracellular D-lactic acid concentration and the D-lactic acid content in the P(LA-3HB) copolymer (Supplemental Fig. S10).

**Table 3:** Comparison of the copolymer strains phaC1Pre_3HB and phaC1437_3HB. The expression of *ldhA* was controlled with the Tet-On system using either 0, 1, 2.5, 5, 6, or 7.5 mg L^−1^ doxycycline. The results represent averages of two or three biological replicates. CDW: Cell dry weight.

Strain	Doxycycline concentration (mg L^−1^)	Total polymer % of CDW	Polymer titer (mg L^−1^)	Polymer yield (mg g^−1^ glucose)	Lactic acid mol %	Ethanol (g L^−1^)	Lactic acid (g L^−1^)	Acetate (g L^−1^)	CDW (g L^−1^)
PhaC1Pre_3HB	0	3.0	56	2.7	17%	9.4	0.10	1.0	1.9
	1	3.5	63	3.0	29%	10.1	0.37	0.9	1.7
	2.5	6.0	85	4.0	72%	8.7	2.2	0.8	1.5
	5	11.5	158	7.5	90%	8.7	3.5	0.6	1.2
	6	14.2	168	8.0	93%	8.9	3.6	0.6	1.3
	7.5	10.4	130	6.2	88%	8.6	3.3	0.7	1.3
PhaC1437_3HB	0	5.0	135	6.4	6%	9.1	0.04	1.7	2.7
	1	5.1	136	6.5	10%	8.7	0.11	1.7	2.7
	2.5	6.2	137	6.5	50%	8.5	1.8	1.4	2.2
	5	17.8	216	10.3	88%	7.9	4.0	0.8	1.4
	6	19.0	232	11.0	88%	7.9	4.3	0.6	1.3
	7.5	9.1	168	8.0	77%	7.1	3.2	1.0	1.8

### 3.4 Molecular weights of the produced PDLA and P(LA-3HB) polymers

In order to compare the molecular weights of the PDLA and P(LA-3HB) produced with the PHA synthases phaC1Pre and phaC1437_Ps6-19_, the strains phaC1Pre_1x, phaC1437_1x, phaC1Pre_3HB, and phaC1437_3HB were grown for 72 h with 20 g L^−1^ glucose and 6 mg L^−1^ doxycycline. The molecular weights of the extracted polymers from both PDLA strains and from the strain PhaC1Pre_3HB were very similar at 24 h, in the range of weight average molecular weight (Mw) 7.1–8.4 kDa and number average molecular weight (Mn) 5.6–6.4 kDa (Table 4, Supplemental Fig. S11). However, the Mw and Mn of the copolymer P(LA-3HB) produced by the strain phaC1437_3HB were approximately 60% and 40% higher than with the other copolymer strains. The narrow dispersity (Đ) below 1.4 indicates that variation in molecular weights within each sample was small. The growth and metabolite results of this experiment are presented in Supplemental Fig. S12.

**Table 4:** The results from the experiment where the strains phaC1Pre_1x, phaC1437_1x, phaC1Pre_3HB, and phaC1437_3HB were grown with 6 mg L^−1^ doxycycline for 72 h. The results represent averages of two or three biological replicates. LA: D-lactic acid; CDW: Cell dry weight; Mn: Number average molecular weight; Mw: Weight average molecular weight; Đ: Dispersity: Polydispersity.

Strain	Polymer % of CDW			LA mol% of polymer			Polymer titer (mg L^−1^)		Mn (kDa)	Mw (kDa)	Đ (kDa)
	0 h	24 h	72 h	0 h	24 h	72 h	24 h	72 h	24 h		
phaC1Pre_1x	0.03 ± 0.02	3.6 ± 0.16	4 ± 0.09	100%	100%	100%	41	67	6.4 ± 0.30	8.4 ± 1.07	1.3 ± 0.1
phaC1437_1x	0 ± 0	4.6 ± 0.54	4.9 ± 0.45	–	100%	100%	75	78	5.6 ± 0.07	7.1 ± 0.18	1.2 ± 0.05
phaC1Pre_3HB	1.6 ± 0.10	8.7 ± 0.30	11.6 ± 0.88	13%	94%	91%	133	157	6.1 ± 0.13	7.5 ± 0.12	1.3 ± 0.01
phaC1437_3HB	3.9 ± 0.12	10.5 ± 0.32	15 ± 1.42	7%	90%	88%	210	230	8.5 ± 0.33	12.2 ± 0.70	1.3 ± 0.03

## 4 Discussion

In this study we demonstrated that the D-lactic acid content of the P(LA-3HB) copolymer produced *in vivo* can be adjusted from 6 mol% to 93 mol% in the yeast *S. cerevisiae* by controlling expression of the lactate dehydrogenase encoding gene *ldhA* with a modified doxycycline dependent Tet-On expression system. Our results highlight the power of the controlled gene expression in tuning of the polymer composition and improving the overall polymer yield.

The *ldhA* expression correlated well with the doxycycline concentration, reaching the highest levels with the maximum 10 mg L^−1^ of doxycycline used. This expression level was approximately 30% higher than with the strong constitutive *pTDH3* promoter showing the power of the modified Tet-On system combined with eight sTF binding sites and the *ENO1* core promoter. Also, the extracellular D-lactic acid concentration correlated with the increase in *ldhA* expression at doxycycline concentrations from 0 to 7.5 mg L^−1^. Above this doxycycline range, the extracellular D-lactic acid concentrations were similar indicating that possibly other factors such as the *ldhA* mRNA translation efficiency or the pyruvate availability limited the D-lactic acid formation. The amount of the produced extracellular D-lactic acid correlated positively with the D-lactic acid content of the copolymer P(LA-3HB) and with the total PDLA accumulation of the CDW *in vivo,* being the highest at doxycycline concentration of 7.5 mg L^−1^. This result demonstrates that the control of the *ldhA* gene expression offers an effective alternative for the P(LA-3HB) tuning *in vivo,* when compared to other suggested means such as monomer feeding to the culture (Jung et al., 2010; Yang et al., 2010) or control of the oxygen availability (Goto et al., 2019; Yamada et al., 2009, 2010, 2011). In addition to this precise *ldhA* control, the Tet-On method showed an increase in the copolymer accumulation in yeast *S. cerevisiae* when the D-lactic acid content of the polymer increased. This is an opposite result to the earlier *E. coli* studies where the increase in the D-lactic acid content of the P(LA-3HB) copolymer correlated with lower total copolymer accumulation levels. The strains without any doxycycline in the media produced small concentrations of D-lactic acid, leading to 6 mol% and 17 mol% D-lactic acid content in the P(LA-3HB) (Table 3). This background D-lactic acid production could be potentially decreased by lowering the expression of the TetR-VP16 as high expression of sTF has been reported to result in the leakage of Tet-On systems (Roney et al., 2016). This could possibly enable the production of copolymers with even wider range of different D-lactic acid contents, reaching to levels below 6 mol%.

We showed accumulation levels of 5.6% PDLA and 19% P(LA-3HB) of CDW, which are over two and five fold higher, respectively, than reported in the previous studies with yeast (Lajus et al., 2020; Ylinen et al., 2021). The P(LA-3HB) accumulation was increased in the cells from 5% to 19% by increasing the D-lactic acid production *in vivo*. This result highlights the importance of sufficient precursor availability in PHA production in yeast, which has been observed also when PHB homopolymer has been produced in *S. cerevisiae* (Carlson and Srienc, 2006; Kocharin et al., 2012, 2013; Kocharin and Nielsen, 2013). We also studied the effect of higher expression of genes encoding PHA synthases. However, we did not observe any significant increase in PHA polymer accumulation by increasing the copy number the PHA synthase genes. In addition, the PDLA and P(LA-3HB) producing strains polymerized only 1.8–2.2% and 3.3–5.2% of the available D-lactic acid, respectively, and rest of the produced D-lactic acid was exported from the cells (Supplemental Table S3). Further studies are required to confirm the effect of the acetyl-CoA availability and the activity of the propionyl-CoA transferases.

The PDLA and P(LA-3HB) polymerization in *S. cerevisiae* strains was studied with two PHA synthases phaC1437_Ps6-19_ and phaC1Pre originating from different *Pseudomonas* species, but carrying the same four amino acid substitutions, E130D, S325T, S477G, and Q481K. These synthases were chosen for comparison since they have shown efficient D-lactic acid polymerization in *E. coli* (Yang et al., 2011) and in our previous study in *S. cerevisiae* (Ylinen et al., 2021). However, the results from *E. coli* study suggest that these two enzymes might have different activities towards D-lactyl-CoA as expression of phaC1437_Ps6-19_ and phaC1Pre resulted in approximately 50 and 65 mol% LA proportions in P(LA-3HB), respectively (Yang et al., 2011). The two PHA synthases showed indeed differences in copolymer P(LA-3HB) formation also in this study in *S. cerevisiae*. The copolymer strain with phaC1437_Ps6-19_ produced approximately 50% longer copolymers (Mw 12.2 kDa) with 5 mol% lower D-lactic acid content (88 mol% LA), than strain with phaC1Pre (Mw 7.5 kDa, 93 mol% LA). This correlation between higher D-lactic acid content and lower molecular weight in P(LA-3HB) copolymer was also observed in other P(LA-3HB) studies (Jung et al., 2010; Yamada et al., 2011). A recent *in vitro* polymerization study, which was carried out with PHA synthase phaC1_Ps_STQK from *Pseudomonas* sp. 61-3, proposes, that the high Tg of PDLA homopolymers (60 °C) inhibits PDLA polymer elongation by a PHA synthase when the PDLA polymer reaches molecular weight of approximately 3 kDa (Matsumoto et al., 2018). Even though several *in vivo* studies demonstrated the production of PDLA homopolymers with molecular weights of up to 30–55 kDa (Mw) (Jung et al., 2010; Lajus et al., 2020; Yang et al., 2011), none of the reported PDLA polymers reached high molecular weights above 1000 kDa (Mw), which are common for the PHB polymers (Fei et al., 2016; Meixner et al., 2018). According to the theory presented in the *in vitro* study, decrease in D-lactic acid content would result in lower Tg of the polymer and thus allow the polymer elongation to continue to higher molecular weights (Matsumoto et al., 2018).

## 5 Conclusions

Sustainable production of biosynthetic polymers with tunable and novel properties will become increasingly important in the future. New ways for optimizing production levels of the biopolymers and controlling their copolymer compositions are needed. In this study we showed how the modified Tet-On method enables tunable control of expression of the lactate dehydrogenase encoding gene *ldhA* in the yeast *S. cerevisiae,* which leads to control of formation of the D-lactic acid monomer *in vivo*. This in turn enabled us to improve production of the homopolymer PDLA and the copolymer P(LA-3HB), and most importantly to adjust the D-lactic acid content in the copolymer P(LA-3HB) from 6 mol% to up to 93 mol%, as a linear response to the *ldhA* expression. The synthetic transcription factor of the Tet-On system responds to the doxycycline levels in the medium. Thus, it provides an easy way to examine the effects and optimal levels of gene expression that are needed for strain improvement and for desired copolymer structures. The system reduces the need for strain constructions such as creating separate strains with different promoter strengths. It can be applied also for other genes within the polymer synthesis pathways, not only for controlling D-lactic acid synthesis *in vivo,* as successfully demonstrated in this work.

## Funding

This work was supported by the 10.13039/501100004157Maj ja Tor Nesslingin säätiö [grant number 201800005]; 10.13039/501100004022Jenny and Antti Wihuri Foundation (for the Center for Young Synbio Scientists); and 10.13039/501100003125Suomen kulttuurirahasto [grant number 00201193]; and 10.13039/501100002341Suomen Akatemia [grant numbers 310191].

## Author contributions

Anna Ylinen: Conceptualization, Methodology, Investigation, Formal analysis, Writing - Original Draft, Visualization, Funding acquisition.

Laura Salusjärvi: Conceptualization, Methodology, Supervision, Writing - Review & Editing. Mervi Toivari: Conceptualization, Supervision, Writing - Review & Editing.

Merja Penttilä: Conceptualization, Supervision, Writing - Review & Editing, Funding acquisition.

## Declaration of competing interest

The authors declare that they have no known competing financial interests or personal relationships that could have appeared to influence the work reported in this paper.
