# Supplementary material for: Control of D-lactic acid content in P(LA-3HB) copolymer in the yeast Saccharomyces cerevisiae using a synthetic gene expression system
Source: Metab Eng Commun. 2022 Apr 30;14:e00199. doi: 10.1016/j.mec.2022.e00199 (PMC9095885; doi:10.1016/j.mec.2022.e00199)
Supplement: Multimedia component 1 [file mmc1.pdf]

Supplementary Materials

Manuscript: "Control of D-lactic acid content in P(LA-3HB) copolymer in the yeast Saccharomyces cerevisiae using a synthetic gene expression system"

Anna Ylinen, anna.ylinen@vtt.fi [1], corresponding author

Laura Salusjärvi, laura.salusjarvi@vtt.fi [1]

Mervi Toivari, mervi.toivari@vtt.fi [1]

Merja Penttilä, merja.penttila@vtt.fi [1, 2]

[1] VTT Technical Research Centre of Finland Ltd. P.O. Box 1000, FI-02044 VTT, Finland

[2] Department of Bioproducts and Biosystems, School of Chemical Engineering, Aalto University, P.O. Box 11000, FI-00076 AALTO, Finland

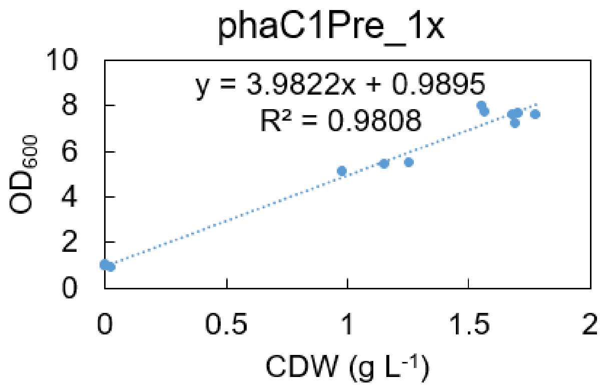

Supplemental Figure S1. Correlation graphs of measured OD_{600} and cell dry weight (CDW) values of strain phaC1Pre_1x. Values align in the linear function of y = 3.9822x + 0.9895, where x represents the CDW and y the OD_{600}, with high R squared value of 0.9808.

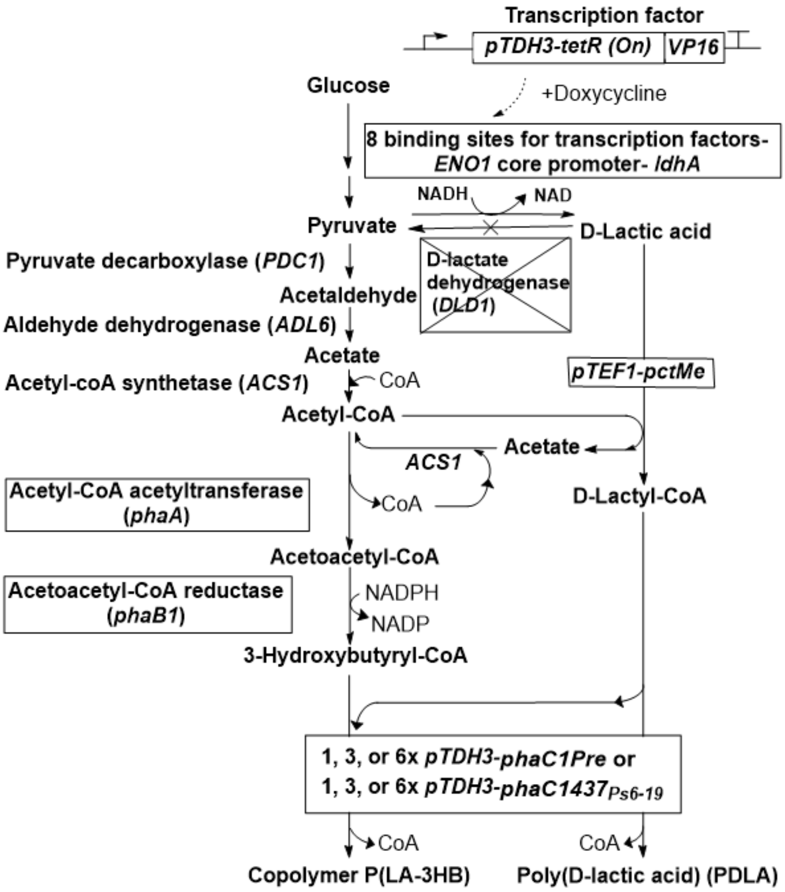

Supplemental Figure S2. Engineered pathways for poly(D-lactic acid) (PDLA) and copolymer P(LA-3HB) production. Genetic modifications are written in boxes.

Supplemental Table S1. The growth of the *S. cerevisiae* strains with doxycycline concentrations of 0-10 mg L^{-1} during the 15 h cultivation experiment in the Bioscreen C equipment.

Strain	Doxycycline (mg L^{-1})	Specific growth rate (h^{-1})	Max OD_{600}
pTEF1-pctMe-TetOn-ldhA	0	0.19	1.64
pTEF1-pctMe-TetOn-ldhA	1	0.16	1.58
pTEF1-pctMe-TetOn-ldhA	5	0.15	1.49
pTEF1-pctMe-TetOn-ldhA	10	0.12	1.23
pTEF1-pctMe	0	0.21	1.70
pTEF1-pctMe	10	0.19	1.71
pTDH3-ldhA	0	0.11	1.22
pTDH3-ldhA	10	0.08	0.96
pTDH3-tetR-VP16	0	0.21	1.70
pTDH3-tetR-VP16	10	0.19	1.57
CEN.PK111-9A	0	0.21	1.70
CEN.PK111-9A	10	0.20	1.68

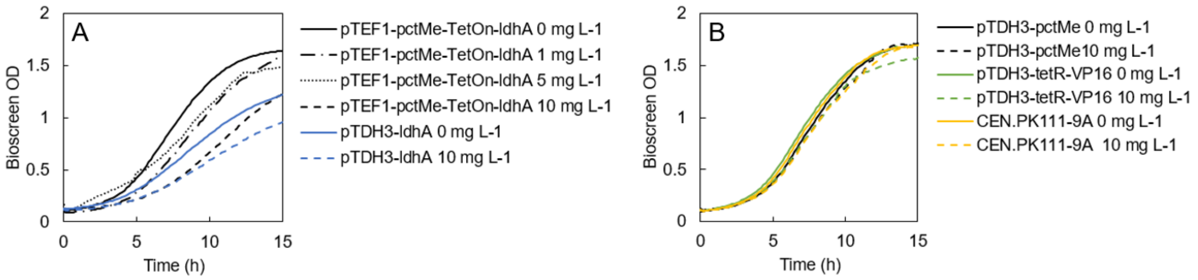

Supplemental Figure S3. The cell growth as OD measured with the Bioscreen C equipment every 10 minutes. The values represent average values of the three biological and technical replicates.

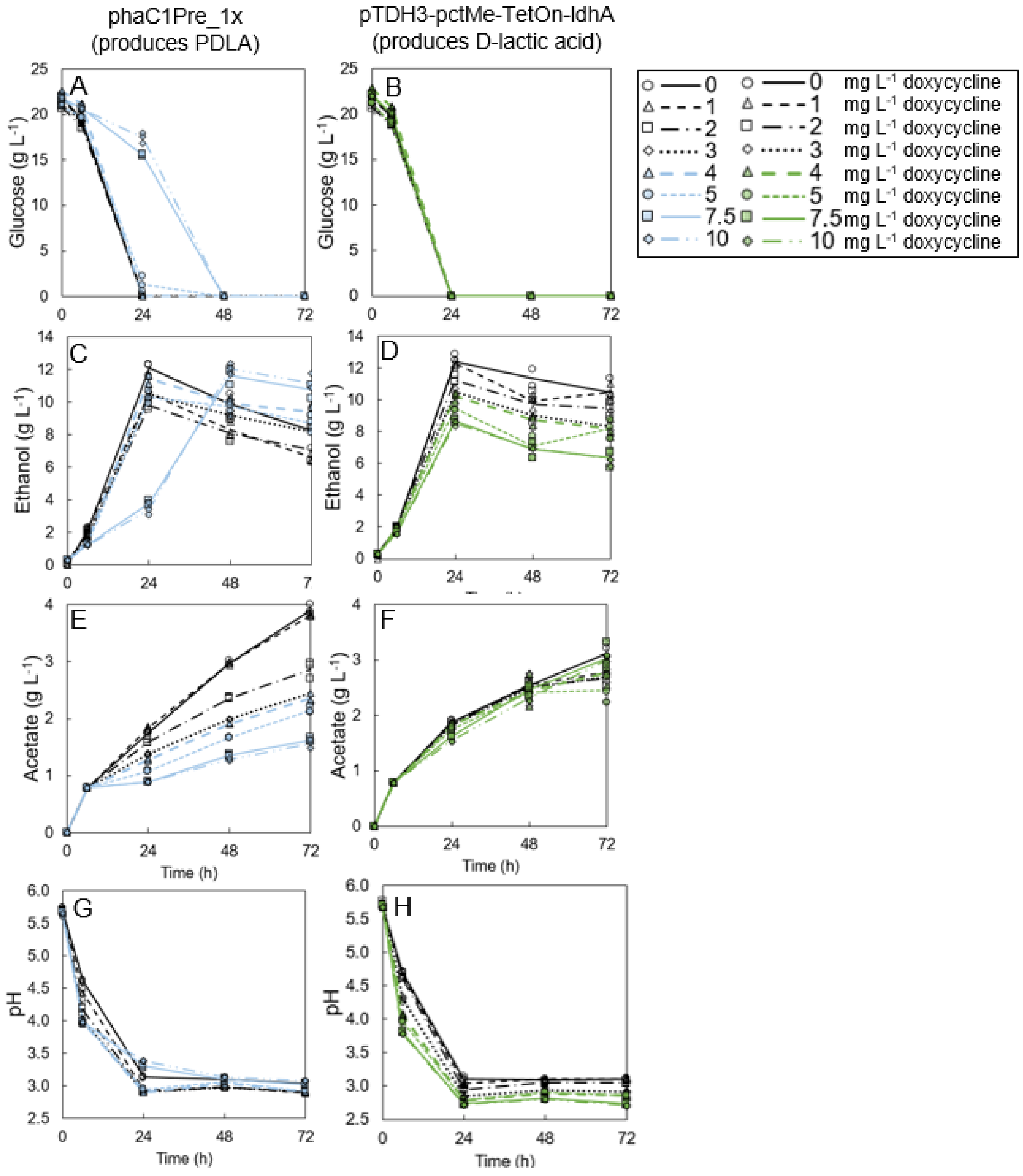

Supplemental Figure S4. The additional data of the cultivation of strains phaC1Pre_1x and control strain pTEF1-pctMe-TetOn-ldhA for 72 h with 20 g L^{-1} glucose and with doxycycline concentrations of 0, 1, 2, 3, 4, 5, 7.5, and 10 mg L^{-1}, presented in Figure 2. A-H: Glucose consumption, and ethanol and acetate production (g L^{-1}), and pH. The values represent averages of the three biological replicates. The individual data points are presented with triangles, squares, diamonds, and circles.

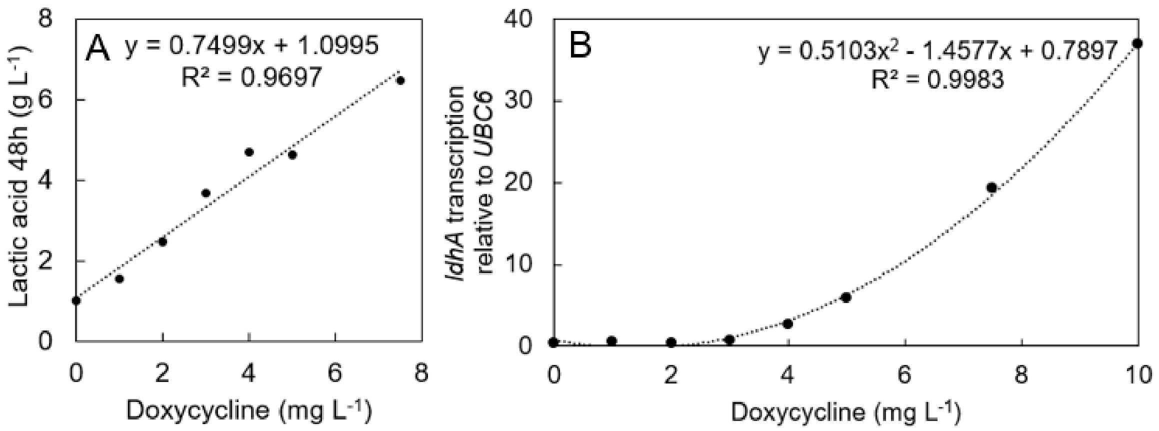

Supplemental Figure S5. Additional graphs for the data presented in Figure 2. A: D-lactic acid production at 48h with different doxycycline concentrations. Values represent averages of three biological replicates. B: The *ldhA* mRNA transcription relative to the ubiquitin-protein ligase gene (*UBC6*) of strain pTDH3-pctMe-TetOn-ldhA with different doxycycline concentrations. Values represent averages of three biological replicates and measured with two oligo pairs (Table 1).

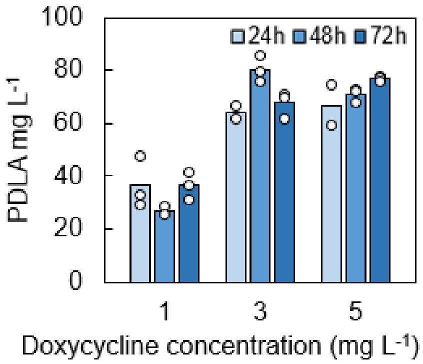

Supplemental Figure S6. PDLA titer of strain phaC1Pre_1x (mg L^{-1}) calculated by converting the measured OD_{600} values to cell dry weight with the correlation function y = 3.9822x + 0.9895 presented in Supplemental Figure S1.

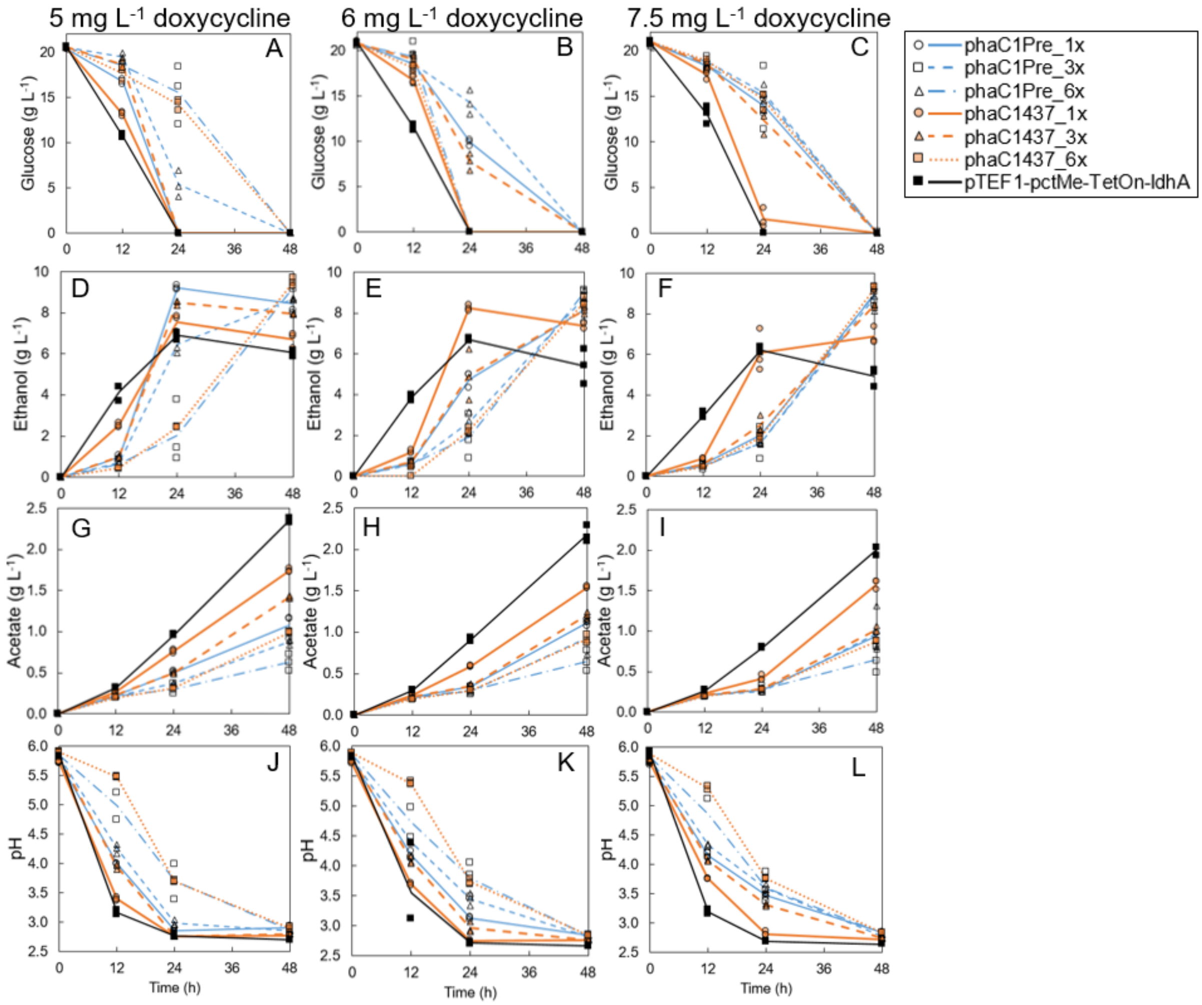

Supplemental Figure S7. The additional data of the cultivation presented in Figure 3. The comparison of the strains with the engineered PHA synthases, phaC1Pre and phaC1437$_{Ps6-19}$, and their expression from one, three, or six copies of the corresponding genes. The *ldhA* expression was controlled with 5, 6, and 7.5 mg L^{-1} doxycycline. A-C: Glucose consumption (g L^{-1}), D-I: Ethanol and acetate formation (g L^{-1}), J-L: Media pH. The values represent the three biological replicates. The individual data points are presented with circles, squares, and triangles.

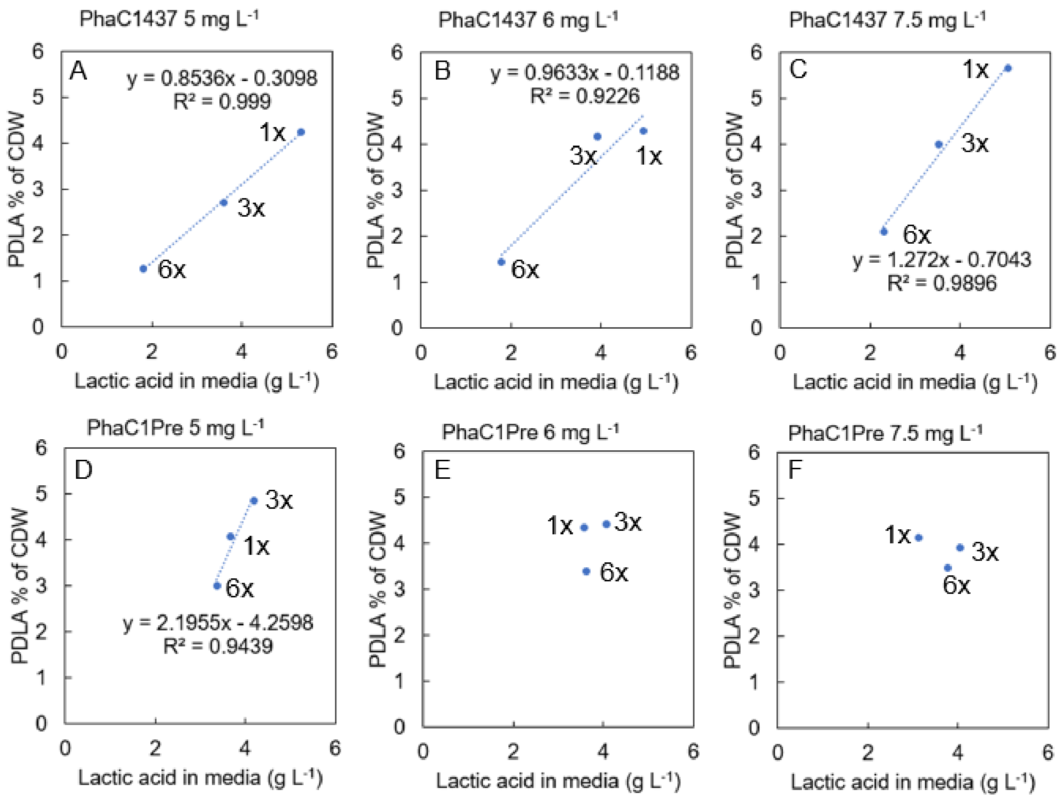

Supplemental Figure S8. The correlation of the D-lactic acid concentration produced to media (g L^{-1}) to the PDLA accumulation in the cells as % of CDW in the strains expressing one, three, or six copies PHA synthase *phaC1437$_{Ps6-19}$* or *phaC1Pre* genes. A, D: The *ldhA* gene expression was controlled with 5 mg L^{-1} doxycycline, B, E: The *ldhA* gene expression was controlled with 6 mg L^{-1} doxycycline, C,F: The *ldhA* gene expression was controlled with 7.5 mg L^{-1} doxycycline.

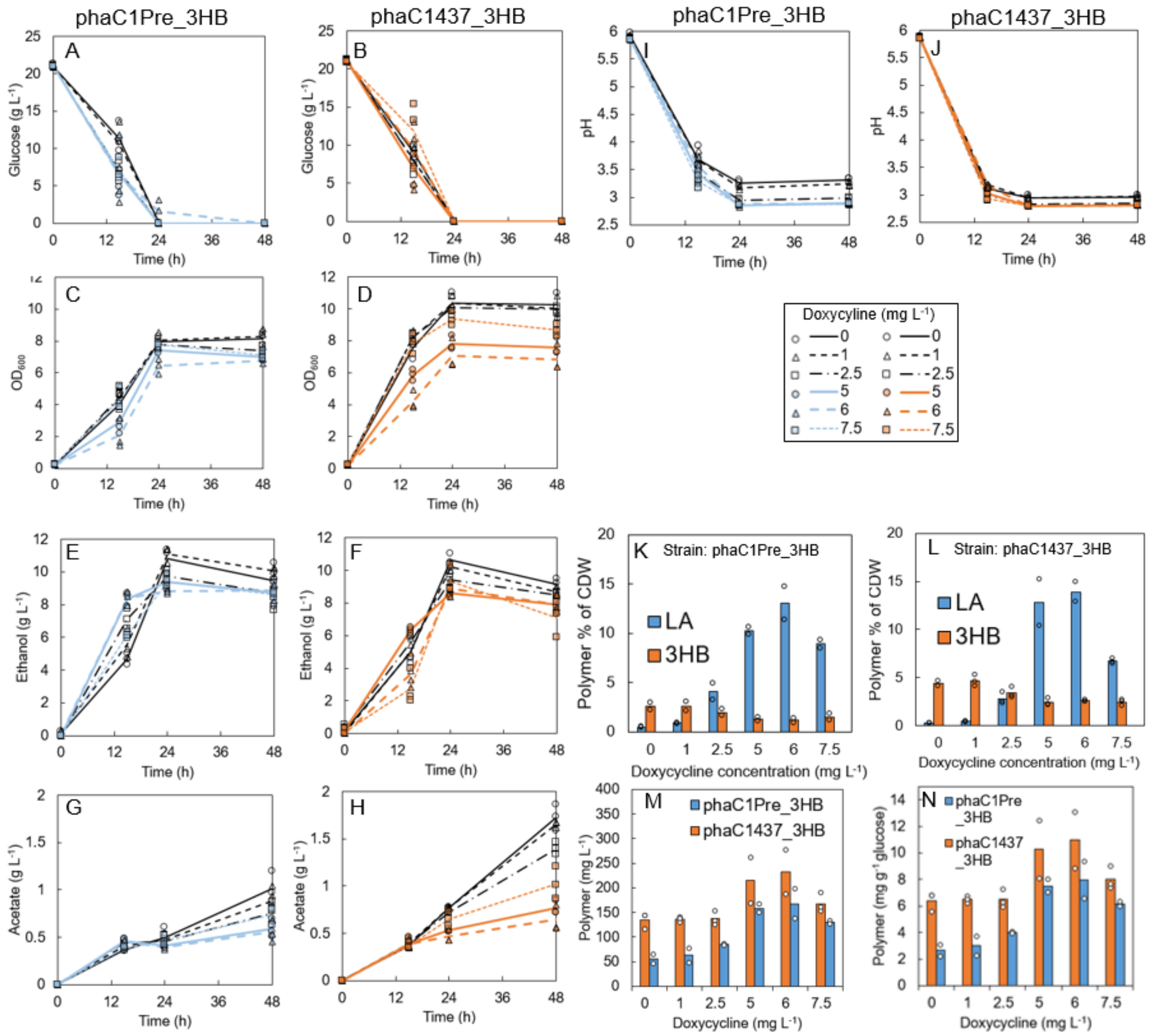

Supplemental Figure S9. The additional data of the cultivation presented in Figure 4. The comparison of the copolymer strains phaC1Pre_3HB and phaC1437_3HB. The expression of *ldhA* rate was controlled with the Tet-On system using 0, 1, 2.5, 5, 6, or 7.5 mg L^{-1} doxycycline. The values represent two or three biological replicates. The individual data points are presented with circles, triangles, or squares. K-L: The bars represent fraction of each monomer of CDW as g g^{-1}.

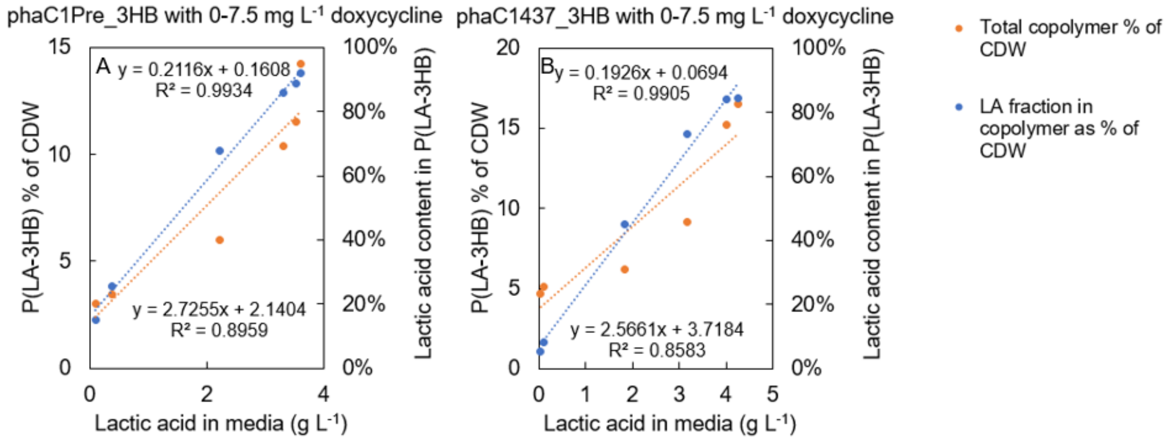

Supplemental Figure S10. The correlation of the D-lactic acid concentration produced into the media (g L^{-1}) to the D-lactic acid content in the copolymer P(LA-3HB) with the copolymer strains PhaC1Pre_3HB and PhaC1437_3HB. The data is also presented in Table 3.

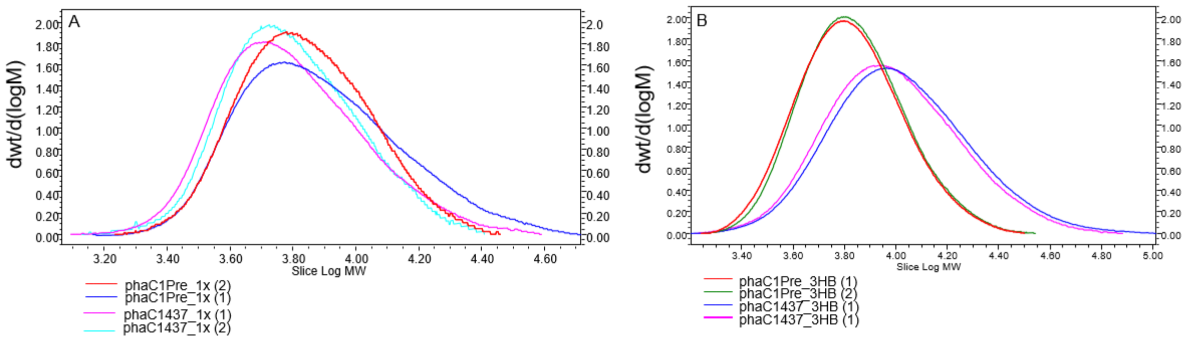

Supplemental Figure S11. The SEC chromatograms of the extracted PDLA (A) and P(LA-3HB) (B) polymers.

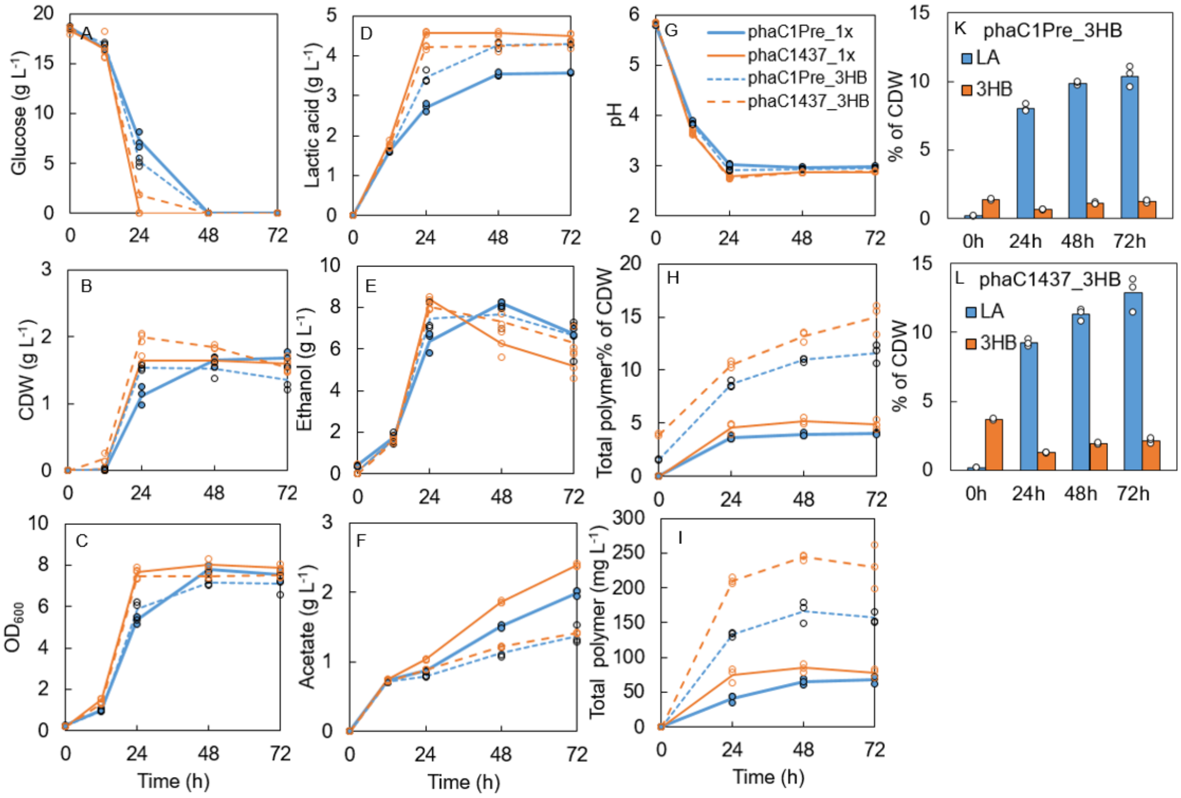

Supplemental Figure S12. The additional data of the cultivation presented in Table 4. The best producing strains were grown with 6 mg L^{-1} doxycycline for 72 h. The results represent averages of two or three biological replicates. K, L: The bars represent fraction of each monomer of CDW as g g^{-1}.

Supplemental Table S3. Comparison of the strains carrying three copies of each PHA synthase. The P(LA-3HB) strains contained additional 3-hydroxyl-CoA pathway (*phaA* and *phaB1* genes). The strains were grown for 48 h with 20 g L^{-1} glucose and 6 mg L^{-1} doxycycline. LA: D-lactic acid

Strain	Results presented in	Polymer	LA yield (C-mol C-mol^{-1} glucose)	Polymerized LA (C-mol C-mol^{-1} glucose)	Polymerized LA (C-mol C-mol^{-1} total LA)
phaC1Pre_3x	a	PDLA	19.7 %	0.43 %	2.1 %
phaC1Pre_3HB	b	P(LA-3HB)	17.1 %	0.80 %	4.5 %
phaC1Pre_3HB	c	P(LA-3HB)	21.7 %	0.74 %	3.3 %
phaC1437_3x	a	PDLA	18.9 %	0.35 %	1.8 %
phaC1437_3HB	b	P(LA-3HB)	20.2 %	1.10 %	5.2 %
phaC1437_3HB	c	P(LA-3HB)	21.3 %	1.03 %	4.6 %

a: Figures 3 and S7
b: Figures 4 and S9
c: Table 4 and Figure S12
